# Cell Deformation by Single-beam Acoustic Trapping: A Promising Tool for Measurements of Cell Mechanics

**DOI:** 10.1038/srep27238

**Published:** 2016-06-08

**Authors:** Jae Youn Hwang, Jihun Kim, Jin Man Park, Changyang Lee, Hayong Jung, Jungwoo Lee, K. Kirk Shung

**Affiliations:** 1 Department of Information and Communication Engineering, Daegu Gyeongbuk Institute of Science & Technology, Daegu, Republic of Korea; 2 NIH Resource Center for Medical Ultrasonic Transducer Technology, Department of Biomedical Engineering, University of Southern California, Los Angeles, CA, USA; 3 Department of Electronic Engineering, Kwangwoon University, Seoul, Republic of Korea

## Abstract

We demonstrate a noncontact single-beam acoustic trapping method for the quantification of the mechanical properties of a single suspended cell with label-free. Experimentally results show that the single-beam acoustic trapping force results in morphological deformation of a trapped cell. While a cancer cell was trapped in an acoustic beam focus, the morphological changes of the immobilized cell were monitored using bright-field imaging. The cell deformability was then compared with that of a trapped polystyrene microbead as a function of the applied acoustic pressure for a better understanding of the relationship between the pressure and degree of cell deformation. Cell deformation was found to become more pronounced as higher pressure levels were applied. Furthermore, to determine if this acoustic trapping method can be exploited in quantifying the cell mechanics in a suspension and in a non-contact manner, the deformability levels of breast cancer cells with different degrees of invasiveness due to acoustic trapping were compared. It was found that highly-invasive breast cancer cells exhibited greater deformability than weakly-invasive breast cancer cells. These results clearly demonstrate that the single-beam acoustic trapping technique is a promising tool for non-contact quantitative assessments of the mechanical properties of single cells in suspensions with label-free.

The mechanical properties of cells play a key role in various cellular functions, such as proliferation, migration, and gene expression^1^,^2^,^3^. Also, they can be altered by diseases or by the external environment^4^. For instance, a red blood cell infected by malaria activates the erythrocytic stages of its life cycle, resulting in the cell’s progressive stiffening. Therefore, the stiffness of a red blood cell can be used for the determination of malaria infection^5^. Also, the mechanics of cancer cells have been measured to determine cancer cell invasiveness, as highly invasive cancer cells are typically softer than weakly invasive cancer cells, allowing them to migrate more easily^6^. As a result, the mechanical properties of a cell can serve as useful biomarkers for the detection of various diseases and in identifications of cell phenotypes, necessitating the development of biophysical tools to quantify cell mechanics.

Many tools capable of probing cell mechanics, including atomic force microscopy (AFM)^7^,^8^, optical tweezers^9^,^10^, and magnetic tweezers^11^,^12^, have been developed. AFM utilizes a nano-sized probe to measure the local stiffness of cells^13^, but it is limited to the measurements of the mechanics of cells with a Young’s modulus greater than 50 Pa. One of its shortcomings is that it requires the probe to be in contact with a cell; moreover, isolation from surrounding vibrations is required to achieve reliable outcomes^7^,^8^. On the other hand, optical tweezers enable one to trap a single cell in a tightly focused laser beam. They have been successfully used to measure the mechanical properties of red blood cells by pulling microspheres attached to these cells^14^. However, they can result in cell damage due to the temperature rise induced by the applied laser^14^. In addition, the trapping force generated by optical tweezers is limited to the pico-Newton range, thus allowing only the trapping of tiny biological samples. Magnetic tweezers have been also shown to be promising for the probing of the mechanical properties of individual molecules, inter-molecular bonds, and whole cells. With this technique, the complex modulus of elasticity of a cell can be quantified and the local viscoelasticity of a cell can be measured^15^. A major drawback of this approach is that spherical magnetic beads of varying diameters must be loaded into the cytoplasm of a cell^16^.

In addition to the tools described above, several ultrasonic techniques have been developed to measure cell mechanics. A high-frequency acoustic-radiation force-impulse microscopic method which works via the photoacoustic detection (PA-ARFI) of a functionalized carbon nanotube attached to the cell membrane was developed to measure cell mechanics^17^. With the PA-ARFI technique, the mechanics of breast cancer cells of different phenotypes can be successfully quantified. A single-beam acoustic trapping technique with a 193 MHz press-focused lithium niobate (LiNbO_3_) transducer was also utilized to study the mechanical properties of a breast cancer cell. In that study, a 5 μm fibronectin-coated polystyrene microbead acoustically trapped was attached to a target cell and was then pulled with acoustic tweezers in order to measure the elastic properties of the cell^18^. Compared to optical tweezers, the single-beam acoustic trapping technique offers several advantages, such as the generation of stronger force of a few nano-Newtons, causing less cell damage, and the use of relatively simple setups, thus demonstrating that it is a promising alternative^19^,^20^.

Although these ultrasonic techniques have the potential to measure cell mechanics, they have a few limitations. First, they need either microbeads or carbon nanotubes to be attached to a target cell, necessitating mastery of the reproduction approach with regard to bead attachment and the uniform distribution and attachment of carbon nanotubes onto the cell membrane. Second, the use of these techniques is limited to the measurement of the mechanical properties of cultured cells. Therefore, they may not be suitable for the measurement of a suspended cell. Note that the suspended state of a cell is more relevant to practical applications of cellular biophysics to medicine^21^. Hence, it would be desirable to develop an ultrasonic technique capable of measuring the mechanical properties of a suspended cell without any materials attached to the cell.

In this paper, we therefore demonstrate a non-contact single-beam acoustic trapping method for the quantification of the mechanical properties of a suspended cell without any materials attached to the cell. The isolation of a suspended target cell from other adjacent cells is achieved by the acoustic trapping of the target cell using a 30 MHz lithium niobate (LiNbO_3_) highly focused ultrasound transducer. In addition, we examine whether a trapped cell is deformed due to the acoustic pressure generated during acoustic trapping and whether the cell deformability due to the acoustic trapping is dependent on its mechanical properties. Furthermore, the deformability levels of breast cancer cells with different degrees of invasiveness were quantitatively compared upon changes in the acoustic trapping force. The results clearly demonstrate the feasibility of the single-beam acoustic trapping technique for quantifying cell mechanics in a non-contact manner without the need for labeling.

## Results

### Cell deformation by acoustic trapping

**Figure 1:**
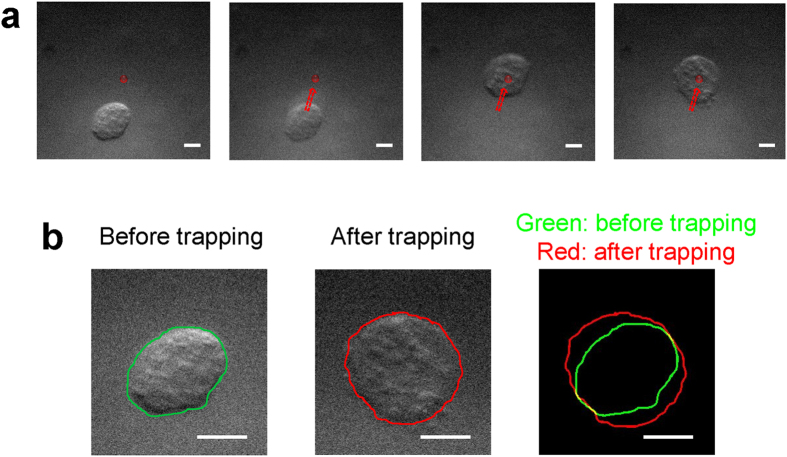
Deformation of a trapped cell due to acoustic trapping: (**a**) acoustic trapping of a breast cancer cell (BT-474). The red solid dot indicates the acoustic beam focus. The dashed arrows represent the direction of movement of a target cell toward an acoustic beam. (**b**) Comparison between the sizes of a cell before and after trapping. The green and red solid lines represent the contour of a trapped cell before and after acoustic trapping, respectively. Input voltage: 22.13 V_pp_ (corresponding acoustic pressure: ~1.64 MPa), number of cycles: 500, PRF: 500 Hz. The scale bars indicate 5 μm.

In order to investigate whether a cell could be deformed due to acoustic trapping, a breast cancer cell was trapped and changes in the cell size in a transverse direction were then monitored using the system described in the method section. Figure 1(a,b) illustrates how the breast cancer cell (BT-474) is moved to the focus and then held by the beam when the trap is switched on [Fig. 1(a) and Supplementary Video 1]. It was found here that the transverse size of a trapped cell becomes larger than that of the cell prior to acoustic trapping. For a detailed examination of the change in the cell size before and after acoustic trapping, the boundaries of the cell before and after acoustic trapping were extracted with ImageJ. As shown in Fig. 1b (right), the cell boundary after acoustic trapping (the red solid line) is larger than that before acoustic trapping (the red solid line). These results clearly demonstrate that the single cell acoustic trapping method results in morphological deformation of the trapped cell under the given condition (1.64 MPa).

### Deformation of a trapped cell with different mechanical properties due to acoustic trapping. 

**Figure 2:**
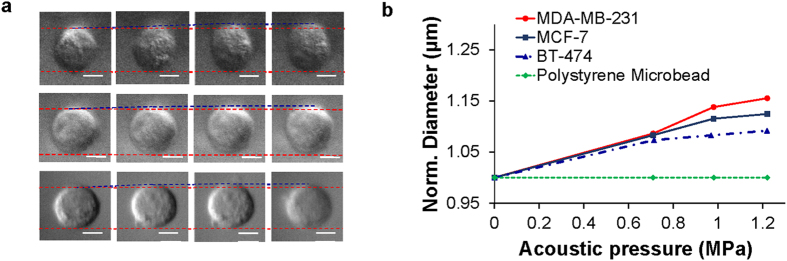
Diameter changes of a MDA-MB-231 cell, a MCF-7 cell, a BT-474 cell, and a polystyrene microbead versus applied acoustic pressures during acoustic trapping. (**a**) Bright-field images of a trapped MDA-MB-231 (upper), MCF-7 (middle), and BT-474 (lower) cell at acoustic pressures of 0, 0.71, 0.97, and 1.22 MPa. (**b**) Measured normalized diameters of trapped samples at the indicated acoustic pressures.

In addition, the diameters of acoustically trapped breast cancer cells, including BT-474 (weakly- invasive), MCF-7 (weakly- invasive), and MDA-MB-231 (highly-invasive) cells, which typically exhibit different mechanical properties, were measured and compared as a function of the acoustic trapping force. Figure 2 illustrates the morphological changes of acoustically trapped cells at the indicated acoustic pressures. The diameters of cells were found to increase as the acoustic pressure was increased (Fig. 2a). The red dotted line indicates the original diameter of the cell before acoustic trapping, whereas the blue dotted line indicates the increased diameter of the cell after acoustic trapping at the given acoustic pressures. The gap between the red and blue dotted line represents the increased length of the diameter of a trapped cell. At 0.71 MPa, the diameters of the trapped cells were observed to be clearly larger than those of the cells before acoustic trapping (0 MPa). As the acoustic pressure was increased further to 1.22 MPa, the diameters of the trapped cells became longer, whereas that of the polystyrene microbead did not change. The normalized diameters of the BT-474 cells were measured and found to be approximately 1.07, 1.08, and 1.09 at 0.71, 0.97, and 1.22 MPa, respectively. The normalized diameters of the MCF-7 cells were measured to be approximately 1.08, 1.11, and 1.12 at 0.71, 0.97, and 1.22 MPa, respectively. In contrast, the corresponding normalized diameters of the MDA-MB-231 cells were approximately 1.09, 1.14, and 1.16 at those acoustic pressures, (Fig. 2b). The normalized diameter of the MDA-MB-231 cell was observed to be larger than that of the MCF-7 and BT-474 cell at 1.22 MPa. These results show that the changes in the diameter of the trapped cells due to acoustic trapping depend on the applied acoustic pressure and on the mechanical properties of an acoustically trapped sample.

### Deformability of highly and weakly invasive breast cancer cells due to acoustic trapping

**Figure 3:**
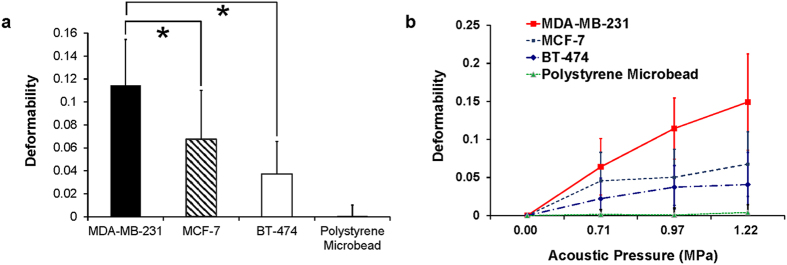
Quantitative analysis of the deformability of breast cancer cells due to acoustic trapping. (**a**) Mean deformability of MDA-MB-231 cells, MCF-7 cells, BT-474 cells and polystyrene microbeads at 1.22 MPa. (**b**) Mean deformability of MDA-MB-231 cells, MCF-7 cells, BT-474 cells, and polystyrene microbeads as a function of the applied acoustic pressure (0, 0.71, 0.97, and 1.22 MPa; sample size: 9). Error bars indicate standard deviations.

A quantitative analysis of the deformability due to acoustic trapping of trapped highly (MDA-MB-231) and weakly invasive (MCF-7 and BT-474) breast cancer cells was carried out in order to investigate whether the single-beam acoustic trapping technique allowed discrimination between mechanical properties of breast cancer cells of different phenotypes. Note that MDA-MB-231 cells are known to be softer than MCF-7 and BT-474 cells^17^. Figure 3 illustrates the change in the deformability of the trapped cells due to acoustic trapping. The MDA-MB-231 cells exhibit greater deformability than the MCF-7 and BT-474 cells and the polystyrene microbeads (control) upon acoustic trapping at 1.22 MPa [p-value = 0.006 (MCF-7) and 0.003 (BT-474) < 0.01]. In addition, the areas of both the highly and weakly invasive breast cancer cells (sample number: 9) increased as the acoustic pressure increased, whereas that of the trapped polystyrene microbeads did not increase. The mean deformability values of MDA-MB-231 cells here were found to be 0.064, 0.114, and 0.149. In contrast, the mean deformability values for the MCF-7 cells were ~0.046, ~0.050, and ~0.068 at acoustic pressure levels of 0.71, 0.97, and 1.22 MPa and the mean deformability values for the BT-474 cells were ~0.022 ~0.047, and ~0.041 at acoustic pressure levels of 0.71, 0.97, and 1.22 MPa, respectively. The changes in the area of the polystyrene microbeads due to acoustic trapping were negligible. These results thus demonstrate that the single-beam acoustic trapping technique may be a promising tool for quantifying the mechanical properties of a suspended cell, as it allows discrimination between highly and weakly invasive breast cancer cells by quantifying the deformability of these cells due to acoustic trapping.

### Changes in cell viability due to acoustic trapping

**Figure 4:**
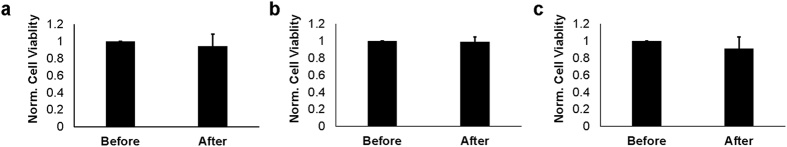
Changes in the viabilities of MDA-MB-231, MCF-7, and BT-474 cells by acoustic trapping: (**a**) mean viability of a MDA-MB-231 cell before and after acoustic trapping (n = 10). (**b**) Mean viability of a MCF-7 cell before and after acoustic trapping (n = 10). (**c**) Mean viability of a BT-474 cell before and after acoustic trapping (n = 10). Error bars indicate standard deviations. The cell viability values were normalized by the calcein fluorescence intensity of the cells before acoustic trapping.

Finally, the viability of breast cancer cells was compared before and after acoustic trapping at 1.22 MPa. Figure 4 illustrates the normalized mean viability of each type of acoustically trapped breast cancer cell before and 30 minutes after acoustic trapping. It was found that the viability levels of MDA-MB-231, MCF-7, and BT-474 cells 30 minutes after acoustic trapping were slightly lower than those of the cells before acoustic trapping, but the difference was not significant (p-values > 0.01). The normalized mean viability levels for the MDA-MB-231, MCF-7, and BT-474 cells were measured to be 0.943 (standard deviation: ±0.1400), 0.991 (standard deviation: ±0.057), and 0.908 (standard deviation: ±0.1406), respectively. Therefore, these results indicate that the cell functions were not significantly compromised by the force exerted during acoustic trapping at the indicated acoustic pressure [p-value = 0.23 (MDA-MB-231), 0.64 (MCF-7), and 0.07 (BT-474) > 0.01].

## Discussion

This paper reports that single-beam acoustic trapping within a certain range of acoustic pressure results in the deformation of an acoustically trapped cell. We clearly observed that a breast cancer cell was deformed due to acoustic trapping at the given acoustic pressures, whereas this was not the case for a polystyrene microbead (Supplementary Video 2). Note that the typical Young’s modulus of a polystyrene microbead is 2.28–3.28 GPa^22^, much greater than that of a breast cancer cell, which has a known Young’s modulus of a few hundreds of Pa to a few kPa^23^. Therefore, these results clearly indicate that the proposed trapping technique under the given condition leads to morphological deformation in a trapped cell but not in a trapped polystyrene bead, further suggesting that deformation in trapped samples may be dependent on their mechanical characteristics.

We also quantitatively compared the deformability of breast cancer cells of different phenotypes due to acoustic trapping at different acoustic pressures. In this experiment, the applied acoustic pressures were limited to much less than ~1.64 MPa for a quantitative analysis of the deformability of trapped cells, as we observed cell blebbing, which may have been caused mainly by decoupling of the cytoskeleton from the plasma membrane, in several acoustically trapped cells when the cells were trapped in acoustic beams at levels exceeding ~1.64 MPa [Supplementary Video 1]. Hence, we examined the deformability of breast cancer cells due to acoustic trapping at less than 1.22 MPa. We here observed that the area of the trapped cells increased as the applied acoustic pressure was increased to 1.22 MPa. In contrast, the polystyrene microbead with a higher stiffness level did not exhibit any significant changes in its area as the acoustic pressure was increased. These results therefore demonstrate that the deformation during the acoustic trapping was likely dependent on the applied acoustic pressure and on the stiffness of the trapped sample. Moreover, we could discriminate between the mechanical properties of highly (MDA-MB-231) and weakly invasive (MCF-7 and BT-474) breast cancer cells in a suspension by comparing their deformability levels due to acoustic trapping at the given axial acoustic pressure (Fig. 3). Previous reports showed that the Young’s modulus of the MDA-MB-231 cells was found to be 500–750 Pa while Young’s modulus of MCF-7 cells was found to be 800–1300 Pa^23^. In addition, MDA-MB-231 cells exhibited a larger membrane displacement than MCF-7 cells upon ARFI application^17^,^24^, indicating that MDA-MB-231 cells were softer than MCF-7 cells. In this study, the MDA-MB-231 cells exhibited greater deformability than the MCF-7 and BT-474 cells, thus indicating that MDA-MB-231 cells are softer than MCF-7 and BT-474 cells. These results are in good agreement with the outcomes obtained by other ultrasonic^17^,^24^ and atomic microscopic techniques^23^,^24^. Note that the single-beam acoustic trapping technique assessed here does not require additional agents such as microbeads or carbon nanotubes to quantify the mechanical properties of cells compared to earlier ultrasonic techniques. Therefore, the present results demonstrate that the single-beam acoustic trapping technique would be very well suited for contact-free, marker-free measurements of the mechanics of a cell in a suspension.

Among other cell previously developed manipulation tools with similar functions, an optical stretcher has become well established as a contact- and marker-free tool for the measurement of cell mechanics. This method utilizes two dual-opposed, slightly divergent, identical laser beams to trap an object and then stretch it. However, in the optical stretcher, micro-fluidic channels are needed for a high-throughput cell analysis, and relatively high laser powers of ~800 mW for each beam are typically required to realize surface forces up to hundreds of pico-Newtons^25^. In contrast, acoustic trapping with a single highly focused acoustic beam results in cell deformation, which may be caused by the squeezing of the cell due to the applied axial acoustic pressure, unlike the stretching of a cell on an optical stretcher. Moreover, the single-beam acoustic tweezers are capable of generating stronger trapping forces up to several nano-Newtons as compared to an optical stretcher, with a relatively simple and inexpensive setup^19^, suggesting that the acoustic trapping technique may be suitable for manipulation of relatively large and stiff biological samples and for quantifications of the mechanics of such samples.

In our previous study related to cancer cells^18^, the cell function was observed to be intact even 24 hours after acoustic trapping force of several hundred megahertz was exerted onto breast cancer cells. Akin to the results shown in our previous studies, the acoustically trapped cells here were still viable after acoustic trapping for five minutes at 1.22 MPa. In this study, we applied tightly focused ultrasound microbeams to the cells for five minutes in the examination of deformability of highly and weakly invasive breast cancer cells due to acoustic trapping at different acoustic pressures. Therefore, we performed the cell viability test after five-minute ultrasound microbeam application to the cells. Note that ultrasound microbeam application for a few seconds in the acoustic trapping of a cell at the acoustic pressure used was sufficient to deform the cell [Supplementary Video 1]. Thus, the acoustic trapping duration required for the measurement of the cell mechanics could be reduced to much less than five minutes. These results indicate that the mechanical properties of a cell can be measured in a considerably healthy condition with the single-beam acoustic trapping technique at the given acoustic pressure level.

## Conclusions

In this paper, we demonstrate that the acoustic trapping technique has the potential to become a valuable tool with which to quantify the mechanical properties of a cell in a suspension without the need for contact or labeling. It was found that acoustic trapping results in the deformation of a trapped cell, which is likely to be caused by the squeezing of the cell. In addition, the deformation of a trapped cell was found to depend on the applied acoustic pressure and on its mechanical properties. Measurement of the mechanical properties of a single cell has been of increasing interest as part of the effort to gain a better understanding of various cell functions, such as motility, division, and adhesion^26^. In particular, the mechanical characteristics of cancer cells have been shown to be highly correlated with their metastatic potentials in various cases^6^. In previous studies, it was reported that a highly invasive cancer cell exhibited softer mechanical characteristics than a weakly invasive cancer cell^23^. Therefore, this unique acoustic trapping technique may be useful for the determination of the invasion potential of cancer cells given its ability to measure the cancer cell mechanics. However, to measure absolute mechanical properties of a cell by using the technique, it would be needed to calibrate the deformability of a cell due to acoustic trapping with cell-mimic phantoms with different mechanical properties, including Young’s Modulus, Poisson ratio, and etc. The cell mimicking phantoms with different mechanical properties are not available at this time and future efforts will be dedicated to obtain absolute mechanical properties of a cell by the single-beam acoustic trapping technique.

## Methods and Materials

### Cell preparation and materials

MDA-MB-231, MCF-7, and BT-474 human breast cancer cell lines were obtained from ATCC (Manassas, VA) and were maintained in Dulbecco’s modified eagle medium (DMEM) containing 10% fetal bovine serum. Hank’s balanced salt solution (HBSS) was purchased from Invitrogen (Grand Island, NY) to maintain the cells during the cell experiment. A trypsin-EDTA solution (Invitrogen, Grand Island, NY) in which to suspend the cells was prepared.

### Acoustic tweezer system

**Figure 5:**
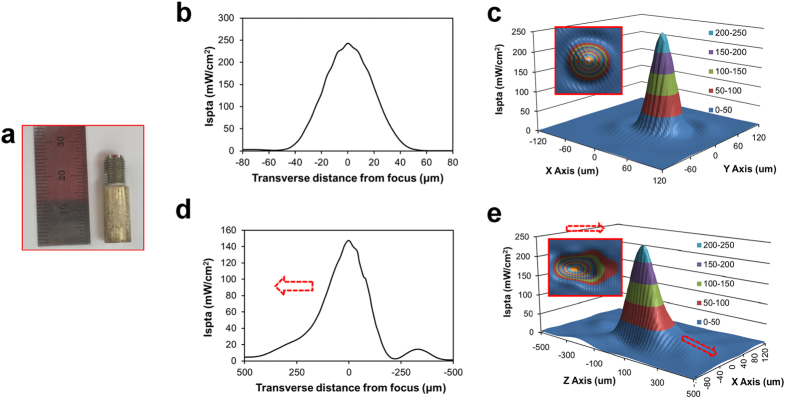
Characteristics of a high frequency ultrasound transducer. (**a**) Photographic image of a transducer (**b**) later beam profile (**c**) two-dimensional ISPTA profile (inset: horizontal section of an acoustic beam) (**d**) axial beam profile (**e**) two-dimensional ISPTA profile. The arrows indicate the direction toward a transducer.

A press-focused LiNbO_3_ transducer at a center frequency of 30 MHz was fabricated with the conventional procedure described previously (Fig. 5a)^27^. For fabrication of the transducer, a 36° rotated Y-cut LiNbO_3_ plate (LiNbO_3_; Boston Piezo-Optics, MA, USA) with a thickness of ~76 μm was first coated with chrome (Cr)/gold (Au) electrodes (Cr/Au; \Nano-Master, TX, USA). The first matching layer was made from a mixture of Insulcast 501 epoxy (Insulcast 501; American Safety Technologies, PA, USA) and 2–3 μm silver particles (Silver; Aldrich Chemical Co., MO, USA) and then lapped to a thickness of 12 μm, followed by the attachment of the layer on one side of the LiNbO_3_ plate. Conductive silver epoxy (E-Solder 3022; Von Roll Isola Inc., USA) was cast on the other side of the LiNbO_3_ for construction of a backing. The LiNbO_3_-matching-backing stack was concentrically placed in the brass housing. The stack and the housing were electrically insulated by filling an epoxy (Epo-Tek 301; Epoxy Technologies, MA, USA) between them. It was press-focused and sputtered with Cr/Au electrodes. A parylene film (14 μm) was then coated over the aperture.

The aperture diameter and focal length of the transducer were 4 mm and 3 mm, respectively (*f*-number = 0.75). The lateral and axial beam widths were measured correspondingly to be 30 μm and 200 μm (−3 dB) with a needle hydrophone (Precision Acoustics, UK), [Fig. 5(b,c)]^28^. In addition, two-dimensional lateral and axial spatial peak temporal average intensity (ISPTA) levels were examined [Frequency: 30 MHz, number of cycles: 10, the pulse repetition frequency (PRF): 500 Hz] [Fig. 5(d,e)]. The acoustic beam intensity was shown to be nearly symmetric at the focal point in the transverse direction [Fig. 5d, inset], whereas the axial beam intensity was found to be slightly non-symmetric [Fig. 5e, inset].

**Figure 6:**
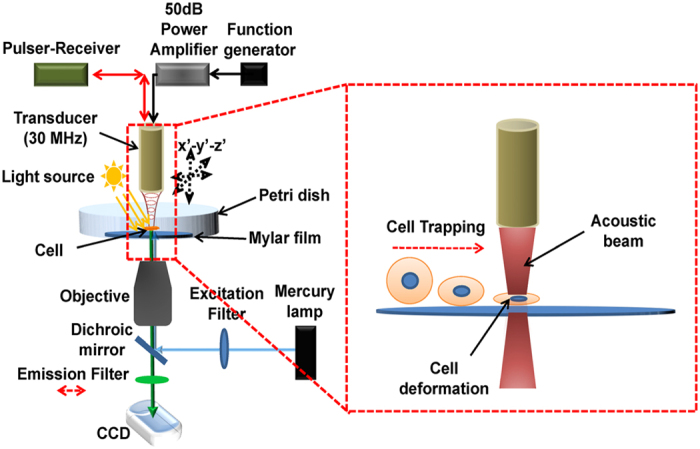
Experimental set-up for the acoustic trapping of a suspended cell. A 30 MHz ultrasound transducer is mounted on a goniometer attached to a 3-axis motorized stage. The transducer is driven by sinusoidal bursts amplified in a 50 dB amplifier for generation of an acoustic beam. The sinusoidal bursts from a function generator are here fed into the 50 dB amplifier. A pulser-receiver is connected to the transducer to adjust acoustic beam focus. For transmission and fluorescence imaging of a target cell in a petri dish during acoustic trapping of the cell, a conventional fluorescence microscope is utilized. A 20× objective lens is utilized for light collection. A DAPI filter set [excitation (EX): 480 nm and emission (em): 530 nm] is utilized for fluorescence imaging. Note that the emission filter is removed when transmission imaging is performed. The red-dotted rectangular indicates conceptual illustrations for cell deformation due to acoustic trapping.

The transducer was mounted on a three-axis motorized stage (LMG26 T50MM; OptoSigma, Santa Ana, CA, USA) equipped with an inverted fluorescence microscope (IX71, Olympus, Center Valley, PA, USA) to align the focus precisely with a target suspended cell by using a pulser-receiver (5910PR; Olympus, Center Valley, PA, USA). In the adjustment of the focus with a target suspended cell, the transducer was focused onto a Mylar film where a target cell was located and then adjusted the tilt of the transducer by examination of echo signal amplitudes from the Mylar film. Note that the echo signal amplitude from the Mylar film became maximal when the transducer was perpendicularly focused on the Mylar film. In order to acoustically trap a target suspended cell, 30 MHz sinusoidal bursts from a function generator (SG382; Stanford Research Systems, CA, USA) were amplified in a 50-dB power amplifier (525LA; E&I ltd., Rochester, USA). The amplified sinusoidal bursts were then input to the transducer. The resultant peak-to-peak (V_*pp*_) voltages of the bursts were adjusted to 0, 4.7, 9.5, and 14.2 V_pp_. The duty cycles were tuned to 500, and PRF was 500 Hz. During the acoustic trapping of the cell, the cell deformation in the lateral direction was monitored and then recorded by a CCD camera (ORCA-Flash 2.8; Hamamatsu Photonics, Hamamatsu, Japan) attached to the microscope (Fig. 6).

### Quantification of the mechanical properties of a trapped cell

Breast cancer cells were plated onto 35mm petri dishes and incubated in the complete medium at 37˚C for 36 hours prior to the cell experiment. The cells were then suspended by the addition of 2ml of the trypsin-EDTA solution into the petri dish after aspiration of the complete medium. This step was followed by the thorough washing of the cells with HBSS. The cells suspended in 2ml of HBSS were added to a 35mm petri dish of which the bottom plate was replaced with Mylar film. A target suspended cell was then isolated from other adjacent cells by acoustic trapping of the cell. Bright-field imaging was conducted to monitor the cell deformation due to acoustic trapping, followed by measurement of the size of the cell in the transverse direction. The size of the cell was also calculated at different acoustic pressures [30 MHz, duty cycles: 500, PRF: 500 Hz, input voltages to the transducer: 0, 4.7, 9.5, and 14.2 V_pp_ (corresponding acoustic pressures: 0, 0.71, 0.97, and 1.22 MPa)].

### Calculation of the deformability of a trapped cell

The deformability (D) was calculated according to the following equation,


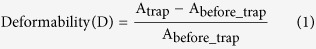


where A_trap_ and A_before_trap_ represent the measured area of the cell after and before the acoustic trapping, respectively. For a detailed examination of the changes in the deformability of the cells before and after acoustic trapping, the area of the cells was precisely quantified using the ImageJ software package (NIH, Bethesda, Maryland, USA).

### Cell viability test

Cell viability tests before and after the acoustic trapping of a target cell were carried out in order to examine whether the applied acoustic trapping force compromised the trapped cell. For each cell viability test, a cell-permeant dye, Calcein AM (Invitrogen, Carlsbad, CA, USA) was loaded into the cell as described previously^18^. After the loading of the dye into the cells, acoustic trapping of the target cell was then carried out for five minutes. For an examination of changes in the cell viability after acoustic trapping, fluorescence imaging of the cell (Ex: 480 nm, Em: 530 nm) was performed before and 30 minutes after the acoustic trapping of the cell. For a statistical analysis, the mean and standard deviation of the fluorescence level at each time interval were obtained with a sample size of n = 10. A two-tailed paired t-test was carried out with the level of significance set as follows: *p-value* < 0.01.

## Additional Information

**How to cite this article**: Hwang, J. Y. *et al.* Cell Deformation by Single-beam Acoustic Trapping: A Promising Tool for Measurements of Cell Mechanics. *Sci. Rep.* **6**, 27238; doi: 10.1038/srep27238 (2016).

## Supplementary Material

Supplementary Information

Supplementary Video 1

Supplementary Video 2

## References

[b1] Deguchi S. & Sato M. Cellular mechanotransduction: diverse perspectives from molecules to tissues. 1st edn, (eds Mofrad M. R. K. *et al.*) 220–233 (Cambridge University Press, 2010).

[b2] Engler A. J. *et al.* Cell Mechanics. Vol. 83 (eds Wang Y. L. *et al.*) 522–544 (Academic Press, 2007).

[b3] Titushkin I. & Cho M. Regulation of cell cytoskeleton and membrane mechanics by electric field: role of linker proteins. Biophysical journal 96, 717–728, doi: 10.1016/j.bpj.2008.09.035 (2009). 19167316 PMC2716454

[b4] Lee G. Y. & Lim C. T. Biomechanics approaches to studying human diseases. Trends in biotechnology 25, 111–118, doi: 10.1016/j.tibtech.2007.01.005 (2007). 17257698

[b5] Hou H. W. *et al.* Deformability based cell margination–a simple microfluidic design for malaria-infected erythrocyte separation. Lab Chip 10, 2605–2613, doi: 10.1039/c003873c (2010). 20689864

[b6] Xu W. *et al.* Cell stiffness is a biomarker of the metastatic potential of ovarian cancer cells. PLoS One 7, e46609, doi: 10.1371/journal.pone.0046609 (2012). 23056368 PMC3464294

[b7] Rico F. *et al.* Probing mechanical properties of living cells by atomic force microscopy with blunted pyramidal cantilever tips. Phys Rev E Stat Nonlin Soft Matter Phys 72, 021914, doi: http://dx.doi.org/10.1103/PhysRevE.72.021914 (2005). 16196611 10.1103/PhysRevE.72.021914

[b8] Titushkin I. & Cho M. Modulation of cellular mechanics during osteogenic differentiation of human mesenchymal stem cells. Biophys J 93, 3693–3702, doi: 10.1529/biophysj.107.107797 (2007). 17675345 PMC2072058

[b9] Titushkin I. & Cho M. Distinct membrane mechanical properties of human mesenchymal stem cells determined using laser optical tweezers. Biophys J 90, 2582–2591, doi: 10.1529/biophysj.105.073775 (2006). 16399828 PMC1403190

[b10] Zhang H. & Liu K. K. Optical tweezers for single cells. J R Soc Interface 5, 671–690, doi: 10.1098/rsif.2008.0052 (2008). 18381254 PMC2408388

[b11] Kasza K. E., Vader D., Köster S., Wang N. & Weitz D. A. Magnetic twisting cytometry. Cold Spring Harb Protoc 2011, 421–422, doi: 10.1101/pdb.prot5599 (2011). 21460048

[b12] Fabry B., Maksym G. N., Hubmayr R. D., Butler J. P. & Fredberg J. J. Implications of heterogeneous bead behavior on cell mechanical properties measured with magnetic twisting cytometry. J Magn Magn Mater 194, 120–125, doi: 10.1016/S0304-8853(98)00564-2 (1999).

[b13] Raman A. *et al.* Mapping nanomechanical properties of live cells using multi-harmonic atomic force microscopy. Nature nanotechnology 6, 809–814, doi: 10.1038/nnano.2011.186 (2011). 22081213

[b14] Musielak M. Red blood cell-deformability measurement: review of techniques. Clinical hemorheology and microcirculation 42, 47–64, doi: 10.3233/CH-2009-1187 (2009). 19363240

[b15] Kilinc D. & Lee G. U. Advances in magnetic tweezers for single molecule and cell biophysics. Integrative biology: quantitative biosciences from nano to macro 6, 27–34, doi: 10.1039/c3ib40185e (2014). 24263142

[b16] Bausch A. R., Moller W. & Sackmann E. Measurement of local viscoelasticity and forces in living cells by magnetic tweezers. Biophysical journal 76, 573–579, doi: 10.1016/S0006-3495(99)77225-5 (1999). 9876170 PMC1302547

[b17] Hwang J. Y. *et al.* Non-contact acoustic radiation force impulse microscopy via photoacoustic detection for probing breast cancer cell mechanics. Biomedical optics express 6, 11–22, doi: 10.1364/BOE.6.000011 (2015). 25657870 PMC4317122

[b18] Hwang J. Y. *et al.* Cell membrane deformation induced by a fibronectin-coated polystyrene microbead in a 200-MHz acoustic trap. IEEE Trans Ultrason Ferroelectr Freq Control 61, 399–406, doi: 10.1109/TUFFC.2014.2925 (2014). 24569245 PMC4030728

[b19] Lee J. *et al.* Single beam acoustic trapping. Applied physics letters 95, 73701, doi: 10.1063/1.3206910 (2009). 19798424 PMC2755305

[b20] Lee J., Lee C. & Shung K. K. Calibration of sound forces in acoustic traps. IEEE transactions on ultrasonics, ferroelectrics, and frequency control 57, 2305–2310, doi: 10.1109/TUFFC.2010.1691 (2010). PMC3056275 20889418

[b21] Maloney J. M., Lehnhardt E., Long A. F. & Van Vliet K. J. Mechanical fluidity of fully suspended biological cells. Biophysical journal 105, 1767–1777, doi: 10.1016/j.bpj.2013.08.040 (2013). 24138852 PMC3797573

[b22] Callister W. D. & Rethwisch D. G. Materials Science and Engineering: An Introduction. 9th edn, (eds Callister W. D. *et al.*) Ch. 15, 580–587 (Wiley, 2013).

[b23] Swaminathan V. *et al.* Mechanical stiffness grades metastatic potential in patient tumor cells and in cancer cell lines. Cancer research 71, 5075–5080, doi: 10.1158/0008-5472.CAN-11-0247 (2011). 21642375 PMC3220953

[b24] Kang B. J., Yoon C., Park J. M., Hwang J. Y. & Shung K. K. Jitter reduction technique for acoustic radiation force impulse microscopy via photoacoustic detection. Optics express 23, 19166–19175, doi: 10.1364/OE.23.019166 (2015). 26367579 PMC4523556

[b25] Guck J. *et al.* The optical stretcher: a novel laser tool to micromanipulate cells. Biophysical journal 81, 767–784, doi: 10.1016/S0006-3495(01)75740-2 (2001). 11463624 PMC1301552

[b26] Kirmizis D. & Logothetidis S. Atomic force microscopy probing in the measurement of cell mechanics. International journal of nanomedicine 5, 137–145, doi: http://dx.doi.org/10.2147/IJN.S5787 (2010). 20463929 10.2147/ijn.s5787 PMC2865008

[b27] Cannata J. M., Ritter T. A., Chen W. H., Silverman R. H. & Shung K. K. Design of efficient, broadband single-element (20-80 MHz) ultrasonic transducers for medical imaging applications. IEEE transactions on ultrasonics, ferroelectrics, and frequency control 50, 1548–1557, doi: 10.1109/TUFFC.2003.1251138 (2003). 14682638

[b28] Huang B. & Shung K. K. Characterization of high-frequency, single-element focused transducers with wire target and hydrophone. IEEE transactions on ultrasonics, ferroelectrics, and frequency control 52, 1608–1612, doi: 10.1109/TUFFC.2005.1516034 (2005). PMC2410096 16285460

