# Supplementary material for: Cell Deformation by Single-beam Acoustic Trapping: A Promising Tool for Measurements of Cell Mechanics
Source: Sci Rep. 2016 Jun 8;6:27238. doi: 10.1038/srep27238 (PMC4897707; doi:10.1038/srep27238)
Supplement: Supplementary Information [file srep27238-s1.pdf]

Cell Deformation by Single-beam Acoustic Trapping: A Promising Tool for Measurements of Cell Mechanics

Jae Youn Hwang[1,*], Jihun Kim[1], Jin Man Park[1], Changyang Lee[2], Hayong Jung[2], Jungwoo Lee[3,*], and K. Kirk Shung[2]

[1]Department of Information and Communication Engineering, Daegu Gyeongbuk Institute of Science & Technology, Daegu, Republic of Korea

[2]NIH Resource Center for Medical Ultrasonic Transducer Technology, Department of Biomedical Engineering, University of Southern California, Los Angeles, CA, USA

[3]Department of Electronic Engineering, Kwangwoon University, Seoul, Republic of Korea

Correspondence and requests for materials should be addressed to J. Y. H. (jyhwang@dgist.ac.kr) or J. L. (jwlee@kw.ac.kr)

Supplementary Video 1. Breast cancer cell deformation due to acoustic trapping.

Supplementary Video 2. Polystyrene microbead deformation due to acoustic trapping.
